# Partial Loss of Inheritable Type I Resistance of Codling Moth to Cydia pomonella granulovirus

**DOI:** 10.3390/v11060570

**Published:** 2019-06-20

**Authors:** Jiangbin Fan, Jörg T. Wennmann, Johannes A. Jehle

**Affiliations:** Institute for Biological Control, Julius Kühn-Institut, Heinrichstraße 243, 64287 Darmstadt, Germany; fan.jiangbin@julius-kuehn.de (J.F.); joerg.wennmann@julius-kuehn.de (J.T.W.)

**Keywords:** *Baculoviridae*, *Betabaculovirus*, pest control, resistance, mass crossing, single-pair crossing, fitness cost

## Abstract

Current knowledge of the field resistance of codling moth (CM, *Cydia pomonella*, L) against Cydia pomonella granulovirus (CpGV) is based mainly on the interaction between the Mexican isolate CpGV-M and CpRR1, a genetically homogeneous CM inbreed line carrying type I resistance. The resistance level of laboratory-reared CpRR1 to CpGV-M was recently found to have decreased considerably, compared to the initially high resistance. To understand the background of this phenomenon, CpRR1 larvae were exposed over several generations to CpGV-M for re-selection of the original resistance level. After five and seven generations of selection, new CpRR1_F5 and CpRR1_F7 lines were established. The resistance ratio of these selected lines was determined by full range bioassays. The CpRR1_F5 strain regained a higher level of resistance against CpGV up to 10^4^-fold based on LC_50_ values compared to susceptible larvae (CpS), which indicated that the absence of virus selection had resulted in a reduction of resistance under laboratory rearing conditions. In addition, some fitness costs of fecundity were observed in CpRR1_F5. Single-pair crossings between CpRR1_F5 or CpRR1_F7 with susceptible CpS moths revealed a dominant but not fully sex-linked inheritance, which suggests a partial loss of previous resistance traits in CpRR1.

## 1. Introduction

The baculovirus Cydia pomonella granulovirus (CpGV) is an important and commercially successful biopesticide agent, which is used to control larvae of codling moth (CM, *Cydia pomonella* L.) in pome fruit production [1,2,3]. In 2003, a CM field population, designated DE-BW-FI03 or CpR, was collected from an organic apple orchard in southwest Germany, where application of commercial CpGV preparations containing the Mexican isolate CpGV-M had failed [1,4]. When individuals of this population were reared in the laboratory for two years on virus-free diets, the established colony still exhibited an at least 100-fold resistance to CpGV-M. After a highly resistant and genetically homogenous inbred-line of CpR, termed CpRR1, was selected by single-pair crossing experiments, backcrossing between individuals of CpRR1 and of susceptible CM strain CpS demonstrated a dominant, sex-linked resistance, which was later also confirmed for CpR [1,5]. In recent years, more than 40 local orchards with CM populations presumed to carry type I resistance have been discovered in Austria, Czech Republic, France, Italy, the Netherlands and Switzerland [6,7,8,9]. It has been suggested that in most of these populations, type I resistance to CpGV-M had developed because resistance-breaking CpGV isolates provided successful control of CM in nearly all orchards with CpGV resistance [10]. 

When the heterogeneous CpR strain was reared for more than 60 generations without virus selection pressure, its resistant level to CpGV-M, based on median lethal concentration values, was still 100-fold higher than that of susceptible CM [11]. Once the CpR strain was exposed to CpGV-M, the resistance level increased up to 1,000,000-fold, which demonstrates that resistant CM larvae have developed a fast and effective adaptive mechanism against CpGV-M with no discernible fitness cost.

Two types of crossing methods, single-pair crossings and mass crossings, were used to select for genetically homogenous resistant CM colonies. CpRR1 was originally established by consecutive single-pair crossings starting with CpR individuals [5]. An alternative approach using successive mass crossings combined with selections on CpGV-M was applied to obtain the strain CpR-CZ from a Czech CM field population [7]. In contrast, the resistant CM strain RGV-8 was generated from eight generations of mass crossings between individuals of a resistant French CM field population and a susceptible CM strain combined with selection of the progeny on CpGV occlusion bodies (OBs) [12]. 

To date, three types of CpGV resistance are known: Type I resistance against CpGV-M is carried by CpRR1, CpR-CZ, RGV-8 and most other CpGV-resistant populations in Europe. Two other forms, type II and type III resistance have been detected recently in Germany [10,13,14,15]. Mass crossing selection of another CM population from North Rhine-Westphalia in Germany, namely DE-NRW-WE-08, resulted in the discovery of the so-called type II resistance, which was shown to be autosomal dominant inherited. Type II resistance is targeted against isolates other than the CpGV-M isolate [10]. Two lines, CpR5M and CpR5S, were selected for five generations using 10^5^ OBs/mL of either CpGV-M or CpGV-S in each generation. Both lines were cross-resistant to CpGV-M and CpGV-S suggesting a possibly linked mode of action [14]. Finally, a third resistance type (III) carrying Z-chromosomal and autosomal inheritance traits was discovered, demonstrating that resistance against CpGV is a highly complex phenomenon, which includes at least two mechanisms and inheritance pathways [15].

Recently, resistance tests using a discriminating concentration of 5.8 × 10^4^ OBs/mL, causing >95% mortality in susceptible CM neonates, showed that the neonates of the strain CpRR1 were more sensitive to CpGV-M compared to what had been observed in previous tests [5,16]. Because this type of resistance is assumed to occur in numerous CM field populations, the expression of resistance within the CpRR1 strain after several years of rearing without CpGV pressure is a matter of considerable interest. To regain the high level of resistance in CpRR1, a mass crossing selection process was conducted for seven generations, after attempts at single-pair crossings had failed. The resulting selection lines, CpRR1_F5 and CpRR1_F7 were then back-crossed with CpS and tested for their susceptibility to CpGV-M to confirm the resistance inheritance pattern. Indeed, a not fully sex-linked inheritance, which suggests a partial loss of previous resistance traits in CpRR1, was recorded for the two selection lines.

## 2. Material and Methods

### 2.1. Insects and Virus

Larvae of laboratory CM populations were maintained individually on a semi-artificial diet [17] in autoclavable 50-well chambers (Licefa, Bad Salzuflen, Germany) at 26 °C under a photoperiod of 16:8 h (light:dark). Both the susceptible strain (CpS) and resistant strain (CpRR1, type I resistance) were reared at the Institute for Biological Control of the Julius Kühn-Institut, Darmstadt, Germany. CpRR1, was originally selected from the field population DE-BW-FI03 (= CpR) in 2006 [8]. It was reared for six years without virus selection pressure after an interim single-pair crossing selection in 2011 [16]. OBs of the Mexican strain of CpGV, termed CpGV-M [18], were stored at −20 °C as a stock virus. 

### 2.2. Re-Selection of CpRR1

**Figure 1:**
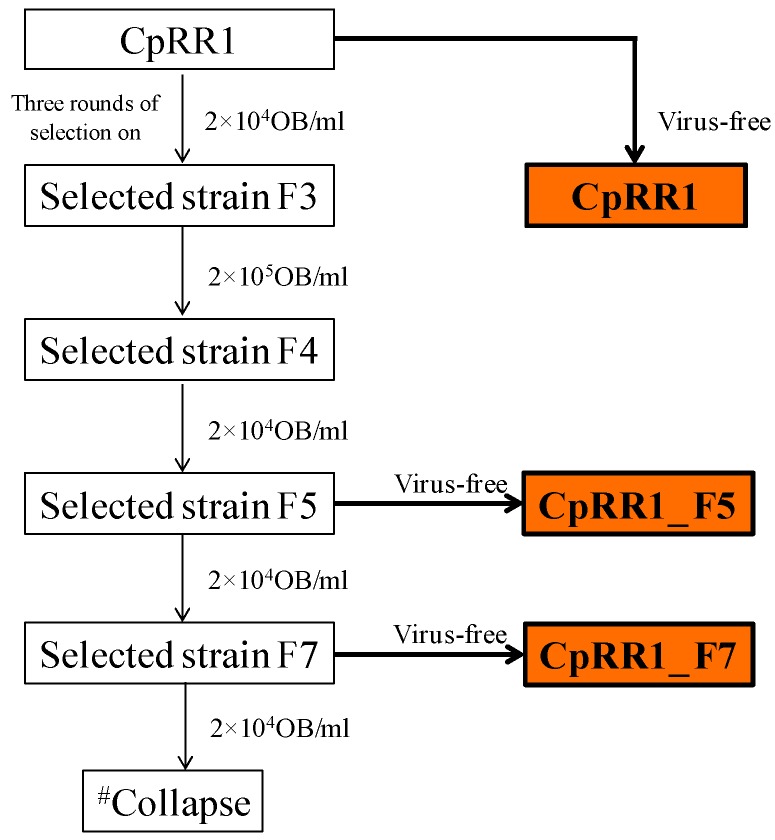
Selection process to regain a high level of resistance of CpRR1. ^#^ Collapse of colony since only a very few male or only female adults survived during selection on CpGV-M.

Re-selection of CpRR1 was initiated by exposing 200 fifth instar (L5) larvae and then 100 larvae at the fourth instar (L4) to a concentration of 2 × 10^4^ OBs/mL of CpGV-M, which were mixed with the artificial diet (for details see [11]). All surviving pupae were pooled and allowed to emerge to adults, which were then mass-crossed [7,12]. Eggs derived from these crosses were incubated to obtain first instar (L1) larvae, which were considered as F1 (generation 1). Then, F1 larvae were reared on a diet containing either 2 × 10^4^ OBs/mL or 2 × 10^5^ OBs/mL of CpGV-M until they developed into adults. After further mass crossing the L1 larvae hatching from the offspring of F1, were used for the next round of selection. Selection was repeated for five generations resulting in CpRR1_F5. A cohort of the F5 larvae was separated for resistance testing and further single-pair crossing experiments. The other cohort of F5 larvae was continued to be reared under virus exposure of 2 × 10^4^ OBs/mL until the seventh generation (CpRR1_F7). About 30–45 larvae of each generation were reared on virus-free diets as a back-up, in case the next round of selection would not provide enough survivors. Then, the backup adults were forwarded to mass crossing and their progeny were further used for the subsequent selection steps (Figure 1).

### 2.3. Bioassays

Full range bioassays were conducted to determine the median lethal concentration (LC_50_) of CpS, CpRR1 and CpRR1_F5. Neonate larvae of the different strains were placed into an autoclavable 50-well box containing a mixture of semi-artificial diet with a series concentration of CpGV-M involving 5 × 10^2^, 1 × 10^3^, 5 × 10^3^, 1 × 10^4^, 5 × 10^4^, 1 × 10^5^ OBs/mL. Groups of 30–40 larvae were prepared for every concentration (for details see [1]). Larvae that died on the first day were assumed to be killed from handling and were therefore excluded in the following analyses. Dead larvae were recorded at 7 and 14 days post-infection (dpi). Each assay was performed independently at least three times. Bioassay data were corrected for untreated control mortality according to the formula of Abbott [19]. Median lethal concentration (LC_50_) was determined by probit analysis using ToxRat Standard Version 2.10 software (ToxRat Solutions GmbH, 2005). 

### 2.4. Single-Pair Crossings

The selected generations CpRR1_F5 and CpRR1_F7 were reared on virus-free diets for at least two generations to obtain sufficient individuals. For single-pair crossings, pupae from susceptible (CpS) and CpRR1_F5 and CpRR1_F7 were separated by sex [1]. After adults emerged, single males of CpS (CpSm) and females of CpRR1_F5 or CpRR1_F7 (CpRR1_F5f or CpRR1_F7f) were transferred to transparent plastic containers for mating and egg deposition. Crossings were also performed with single CpSf×CpRR1_F5m or CpRR1_F7m. Paired adults were fed on a 10% sugar solution in a 35 mm Petri dish. Eggs were collected every two days and stored at 8–10 °C for a maximum of six days [8]. Pooled eggs were incubated at the same conditions as mentioned above. The hatched neonates of each single-pair crossing were then divided into two cohorts, one of which was used for bioassay whereas the other was set as untreated control. Neonate larvae were exposed to the discriminating concentration of 5.8 × 10^4^ OBs/mL, which was previously shown to cause >95% mortality in susceptible CpS larvae at 7 dpi [5]. Mortality was recorded at 7 and 14 dpi and corrected for untreated control mortality according to the formula of Abbott [19].

The number of eggs produced by CpRR1_F5 and CpS in single-pair crossings was counted to evaluate the fecundity. The ratio of hatched neonates to all eggs was considered as representative of fertility. Differences in fecundity and fertility were then used to assess the fitness cost of CpRR1_F5 compared to CpS using Student’s *t*-test after assumption of normal distribution was checked with Shapiro-Wilk´s normality test (R software package version 3.4.4 in RStudio 1.1.442).

## 3. Results

### 3.1. Mass Crossing Selection

Re-selection of CpRR1 larvae to its previous resistance level was achieved by consecutive mass crossings of CpRR1 adults followed by exposure of L1 larvae to CpGV-M OBs. For this, fourth (L4) and early fifth (L5) instars of CpRR1 larvae (CpRR1_F0) were exposed to 2 × 10^4^ OBs/mL of CpGV-M in diet during the rest of larval development (Table 1). Starting from the obtained progeny CpRR1_F1, two rounds of selection followed at the same concentration but with neonate (L1) larvae exposed to virus OBs, resulting in generation of CpRR1_F3. Survival ranged between 22.0% and 7.7% for the different generations (Table 1). Then, the OB concentration was increased to 2 × 10^5^ OBs/mL to select for CpRR1_F4 larvae. As the percentage of surviving adults decreased drastically to only 3.1% for CpRR1_F3, CpRR1_F4 larvae and the following generations CpRR1_F5 to CpRR1_F7 were further selected at the lower virus concentration of 2 × 10^4^ OBs/mL. Eventually, when the CpRR1_F7 population was built up, selection could not be continued because the survival was only 0.6% and either only female or only male larvae developed to adults from four different crossing experiments, which prevented continuation of the selection experiment.

### 3.2. Resistance Ratio

Median lethal concentration (LC_50_) of CpS, CpRR1 and CpRR1_F5 against CpGV-M was determined to compare the susceptibility and the resistance level of the different CM colonies. At 7 dpi, the LC_50_ values of CpGV-M in CpS and CpRR1 neonates were 1.99 × 10^3^ and 1.27 × 10^5^ OBs/mL, respectively. The goodness of fit test (χ^2^ value) of the CpS at 7 dpi indicated considerable deviation from the probit model, although the LC_50_ values at 7 and 14 dpi were very similar to previous experiments, indicating that the CpGV-M sample used in the experiments was fully active against susceptible CMs. In contrast, the LC_50_ in CpRR1_F5 could not be determined due to low larval mortality, but it was estimated from the extrapolated probit line to be >2.0 × 10^7^ OBs/mL. At 14 dpi, the LC_50_ values of CpS, CpRR1 and CpRR1_F5 were 4.5 × 10^2^, 1.60 × 10^4^ and 2.32 × 10^5^ OBs/mL, respectively (Table 2). Thus, the resistance ratio based on LC_50_ values of CpRR1 compared to CpS increased by 64-fold and 36-fold at 7 and 14 dpi, respectively. For CpRR1_F5, the resistance ratio was increased 517-fold at 14 dpi. Since most CpRR1_F5 larvae were still alive at 7 dpi, its estimated resistance ratio compared to CpS was >10,000-fold (Table 2).

### 3.3. Single-Pair Crossing

Resistance testing using the discriminating concentration of 5.8 × 10^4^ OBs/mL resulted in 96.7% mortality at 7 dpi, proving the susceptibility of CpS, whereas CpRR1 and CpRR1_F5 showed a mortality of 40.9% and 49.2%, respectively (Table 3). To further analyze the mode of resistance in CpRR1_F5 and CpRR1_F7 individual, single-pair backcrosses with susceptible CpS followed by resistance testing were performed. When CpRR1_F5 females were crossed with CpS males, mortality was 63.4% at 7 dpi, slightly higher than the expected 50% mortality (Table 3). In CpRR1_F5m × CpSf crosses, induced mortality was 12.1% at 7 dpi, also slightly exceeding the expected 0%. At 14 dpi, mortality increased to 87.2% and 57.4% for CpRR1_F5f × CpSm and CpRR1_F5m × CpSf, respectively, indicating high larval mortality with increasing incubation time. Most of the infected insects died prior to pupation as only two male and seven female pupae were obtained from a total of 638 infected progenies in 11 single-pair crossings.

Induced mortality of offspring from CpRR1_F7f × CpSm was 16.0% and 16.3% at 7 dpi and 14 dpi, respectively, which was much lower than the predicted 50% mortality at 7 dpi. In particular, seven male and ten female larvae survived and developed into pupae in this bioassay, which was also not compatible with the model of a Z-linkage of resistance (Table 3). In the CpRR1_F7m × CpSf crossings mortality was only 3.8% and 4.1% at 7 dpi and 14 dpi, respectively.

### 3.4. Fitness Cost

**Figure 2:**
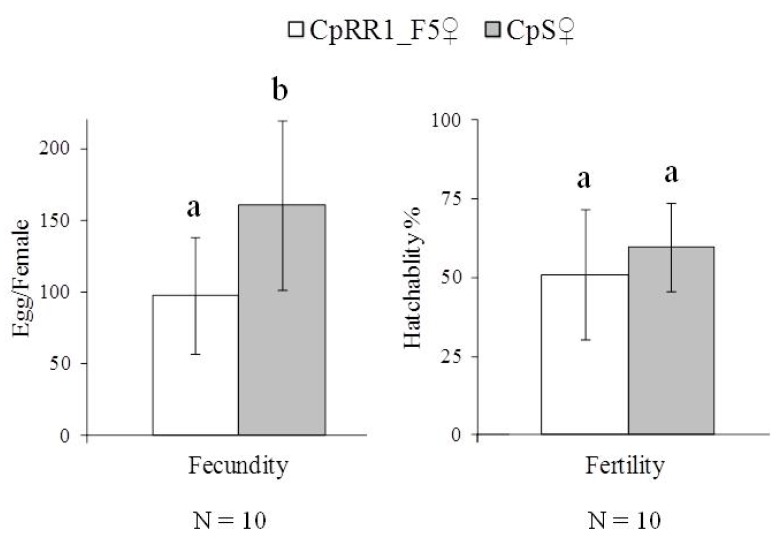
Fecundity and fertility of CpRR1_F5 and CpS. The eggs resulting from each (N = 10) single-pair crossings of CpRR1_F5 × CpRR1_F5 and CpS × CpS, respectively, were counted to estimate their fecundity. The fertility was assessed by the percentage of successfully hatched eggs. Error bars indicate standard deviation (SD). The different letters (a, b) on the top of the SD bar indicate statistically significant differences (*t*-test, *p* < 0.05).

Fecundity of CpRR1_F5 females was significantly lower than that of CpS females when egg production of ten single-pair crossings was quantified (Figure 2) (*t*-test, *p* < 0.05). However, the fertility values of CpRR1_F5f (51.9%) and CpSf (59.8%) were similar (*t*-test, *p* = 0.28). These observations showed that there were some fitness costs in fecundity in the CpRR1_F5 strain when the resistance was partially recovered.

## 4. Discussion

The resistant CM strain CpRR1 was selected in 2006/2007 from the resistant field population CpR [5]. In previous bioassays, CpGV-M did not cause significant mortality in CpRR1 larvae and the LC_50_ was estimated to be 6.92 × 10^8^ OBs/mL and 8.53 × 10^6^ OBs/mL at 7 dpi and 14 dpi, respectively, suggesting a resistance level of 10^4^- to 10^5^–fold [5,16]. Since then, the CpRR1 strain has been reared on semi-artificial diet under virus-free conditions in the laboratory [21]. In 2011, a re-selection was performed with single-pair crossings [16]. When tested in 2015, CpRR1 appeared to be more susceptible to CpGV-M than in previous tests [22]. This finding was corroborated by an LC_50_ determination, demonstrating a considerable decline in the resistance level of the current CpRR1 rearing to only 64-fold and 36-fold resistance in 7- and 14-day bioassays, respectively (Table 2). When CpR was reared for more than 60 generations without CpGV pressure under laboratory conditions, a more or less stable resistance level and no fitness costs of resistance was observed [11]. 

Most laboratory-selected baculovirus-resistant insect colonies become virus sensitive again when the virus selection pressure is removed, most likely because of the fitness costs of carrying the resistance [23]. On the other hand, the resistance of cabbage loopers (*Trichoplusia ni*) against Trichoplusia ni single nucleopolyhedrovirus (TnSNPV) was stable for at least five generations without selection following nine generations of TnSNPV selection [20]. Artificially selected resistance of smaller tea tortrix strain against Adoxophyes honmai nucleopolyhedrovirus (AdhoNPV), harboring a midgut-conferring resistance, showed an over 67,000-fold increased resistance level compared to susceptible larvae; the resistance was stable for more than 168 generations in 13 years of laboratory rearing without further AdhoNPV selection pressure [24,25]. Therefore, it is hypothesized that the CpRR1 strain lost full expression of its original resistance trait because of long-term laboratory rearing without CpGV pressure.

To elucidate the reasons for the decline in resistance and to re-establish a highly resistant CM colony, seven consecutive mass crossings of CpRR1 adults, each followed by selection of progeny larvae on CpGV-M were carried out. The OB concentration used ranged from 2 × 10^4^ OBs/mL to 2 × 10^5^ OBs/mL. After five generations, the selection line CpRR1_F5 was established with an estimated LC_50_ of >2 × 10^7^ OBs/mL at 7 dpi, representing a >10^4^-fold resistance compared to CpS, which was about 35 times lower than that in the original CpRR1 [5]. When mortality after 14 days is considered, the resistance factor of CpRR1_F5 was about 517, again about 20 times lower than the 10^4^-fold level observed in previous experiments. Further selection to induce higher resistance failed when CpRR1_F7 was established because the colony was lost due to high mortality. It cannot be ruled out that inbreeding effects caused the loss of CpRR1_F7. Female adults of CpRR1_F5 produced significantly fewer eggs than CpS females, which could be an effect of inbreeding or caused by fitness costs related to resistance. As shown in Table 1, only a very low number of adults survived during the selection process, suggesting that inbreeding may have reduced the genetic diversity present in the selection lines. Mass crossing selections have been successfully applied to select genetically homogenous CM strains resistant to baculoviruses: the French selection line RGV-8 was established through mass crossings using a discriminating concentration of CpGV-M causing 98.3% mortality of susceptible CM larvae for eight generations [9,26]. The resulting RGV-8 colony, which exhibited a 7000-fold resistance level compared to susceptible CM, showed a strongly dominant, sex-linked resistance pattern, and also suggested that a further resistance-related allele or mode was needed to fully explain the observed results of backcrossing and resistance testing [12]. Similarly, the CM strain CpR-CZ that was selected from the survivors of three mass crossings of resistant individuals collected in the field, showed a dominant, Z-linked inheritance pattern [7]. It is assumed that mass-crossed RGV-8 and CpR-CZ harbor a more heterogeneous genetic background as this was the case for CpRR1, which was developed from a very few individuals. Theoretically, the CpRR1 strain originating from single resistant pairs should not contain susceptible individuals if resistance is stably inherited, even without further selection of resistance. This is apparently not the case [5]. Resistance to CpGV-M in CpRR1_F5 and CpRR1_F7 was regained by selection on CpGV-M, though its resistance level was about 30–50 times lower than that of the original CpRR1 strain (Table 2 and Table 3). The low survival of pupae and low emergence of adults observed during the experiments led to the collapse of the selection line CpRR1-F7, a further indication that the original level of resistance of CpRR1 could not be fully recovered.

According to the well-known mode of type I resistance of CpRR1, dominant resistance factor(s) are located on the Z chromosome and follow classical dominant Mendelian inheritance [5]. Single-pair crossings carried out between the newly established CpRR1_F5 or CpRR1_F7 strains and susceptible CpS larvae as well as among CpRR1_F5 larvae revealed the interesting phenomenon that their resistance did not fully comply with the dominant Z-linked inheritance mode. First, the mortality of progeny of CpRR1_F5f × CpRR1_F5m was nearly 50% after 7 days and was therefore much higher than expected from the LC_50_ determination. Second, the observed mortality in single-pair crossings of CpRR1_F5f × CpSm and CpRR1_F5m × CpSf was 63.4% and 12.1%, respectively, and thus slightly higher than the expected value of 50% and 0%, respectively (Table 3). Although the observed mortality rates were still consistent with a dominant Z-linked inheritance, at a lower resistance level, the finding of two surviving males and five surviving female pupae from the CpRR1_F5m × CpSf crosses cannot be explained by the previous resistance model established by Asser-Kaiser et al. [5], which would have predicted no survival of heterozygous Z^R^Z^S^ males and susceptible Z^S^W females from these crosses. Third, reciprocal single-pair crosses of CpRR1_F7 × CpS resulted in low mortality of only 16.0% and 3.8% after 7 and 14 days, indicating that there was no strong Z-linkage of resistance in this line (Table 3). Strikingly, seven male (expected Z^R^Z^S^) and ten female (expected Z^S^W) pupae survived the experiment, also contradicting the previous resistance model [5]. Thus, these results overturned the sex-linked pattern of resistance because expected Z^S^W female larvae would not survive in the 7-day resistance test, nor would they pupate. Survival of male (Z^R^Z^S^) and female (Z^S^W) pupae in the resistance tests hinted that the resistance factor in the Z chromosome was no longer fixed.

These findings are somewhat reminiscent of the observations made with respect to the resistant CM strain CpRGO (type III resistance) [15]. In that case, no coherent Mendelian pattern of Z-linked or autosomal inheritance was found for resistance to CpGV-M. For CpRGO, the results could be partly explained by a combination of Z-linked and autosomal traits contributing to a heterogeneous response when progeny of different crossings were challenged with CpGV-M. A similar situation appeared again in the CpRR1_F7 after seven rounds of re-selection. Interestingly, despite a strong dominant Z-dependent inheritance controlled by a single major gene, additional factors not subject to the rules of Mendelian inheritance were also observed for the French colony RGV-8 [12].

In conclusion, the type I resistance of CpRR1 is not fully stable and was lost in part during many generations of virus-free rearing. Re-selection recovered resistance; however, this was not at its previous level. Though a dominant resistance mode could be determined after re-selection, its linkage to the Z chromosome could not be fully verified. These findings indicate a certain instability and loss of resistance in CpRR1 as well as potential autosomal factors involved in its expression.

## Figures and Tables

**Table 1:** Survival and adult sex ratio of CpRR1 progeny at each selected generation. The number [N] and instars of larvae used in each selection step, the percentage of surviving pupae and adults of each treatment, and the percentage of resulting males including the numbers of males and females [m:f] of surviving adults are shown.

Generation	N	Larval Instar	CpGV Seletion (OBs/mL)	Surviving Pupae (%)	Surviving Adult (%)	Sex Ratio (% Male) [m:f]
F0	300	L5 + L4	2 × 10^4^	27.3	22.0	53.7 [36:31]
F1	100	L4	2 × 10^4^	11.0	9.0	66.7 [6:3]
F2	326	L1	2 × 10^4^	8.6	7.7	64.0 [16:9]
F3	196	L1	2 × 10^5^	4.1	3.1	16.7 [1:5]
F4	363	L1	2 × 10^4^	17.6	12.4	53.3 [24:21]
F5	95	L1	2 × 10^4^	2.1	2.1	0 [0:2]
F6^a^	92	L1	2 × 10^4^	5.4	5.4	60.0 [3:2]
F7^#^	163	L1	2 × 10^4^	0.6	0.6	collapse [1:0]

F6^a^ was the progeny of F6♀ crossed with F5♂ population. F7^#^ population obtained from surviving larvae in this seventh selection, was used to continue with CpGV-M selection for four generations under the same concentration of 2 × 10^4^ OBs/mL, but there was no-paired adult survival (only male survival adults or only female adult alive) causing collapse of the colony.

**Table 2:** Median lethal concentration (LC_50_) observed for the resistant codling moth strains CpRR1, CpRR1_F5 and the susceptible strain of CpS infected with CpGV-M. Given are the total number (N) of tested L1 larvae, the LC_50_ values at 7 and 14 days post infection (dpi) and their the 95% confidence intervals (CI), the slope and standard error (SE) of the probit line as well as χ^2^ value and degree of freedom (df), as well as the potency (LC_50 resistance strain_/LC_50 CpS_) of the different strains.

Strain	N	7 dpi				14 dpi			
		LC_50_ (95% CI) [OBs/mL]	Slope ± SE	χ^2^ (df)	Potency	LC_50_ (95% CI) [OBs/mL]	Slope ± SE	χ^2^ (df)	Potency
CpS	1075	1.99 (0.98–4.05) × 10^3^	1.26 ± 0.04	30.81 (4)	1	4.50 (2.06–11.90) × 10^2^	1.01 ± 0.04	14.72 (4)	1
CpRR1	1550	1.27 (1.08–1.63) × 10^5^	2.36 ± 0.15	2.37 (4)	64	1.60 (1.41–1.81) × 10^4^	1.97 ± 0.05	6.47 (4)	36
CpRR1_F5	362	>2 × 10^7^	0.45 ± 0.10	3.17 (3)	>10,000	2.32 (0.98–13.22) × 10^5^	0.77 ± 0.09	4.26 (3)	517

n.d., not computable because value is beyond the tested concentrations by more than a factor of 1000; extrapolated estimation of LC_50_ at 7 dpi for CpRR1_F5 was >2.0 × 10^7^ OBs/mL based on probit line.

**Table 3:** Resistance testing of neonate larvae from single-pair crossings of CpRR1_F5 or CpRR1_F7 with CpS exposed to CpGV-M at a discriminating concentration of 5.8 × 10^4^ OBs/mL. Given are the crosses and the number of independent single-pair crossings (N), total number of tested larvae (n), Progeny genotypes by the hypothesis of Z-chromosomal inheritance, mean mortality and standard deviation (SD) after 7 and 14 days post infection (dpi), as well as the sex ratio of pupae; f, female; m, male.

Strain	Crosses	N, nn	Progeny Genotypes, by Z Hypothesis^$^		Observed Mortality (%) at 7 dpi	Observed Mortality (%) at 14 dpi	Pupae
			progeny	Exp. mort. at 7 dpi (%)	Mean ± SD	Mean ± SD	Sex Ratio (% male) [m:f]
CpS	CpSf × CpSm	4, 318	Z^S^Z^S^, Z^S^W	100	96.7 ± 3.9	98.3 ± 3.4	-
CpRR1	CpRR1f × CpRR1m	5, 437	Z^R^Z^R^, Z^R^W	0	40.9 ± 4.3	79.2 ± 8.5	-
CpRR1_F5	CpRR1_F5f × CpRR1_F5m	7, 363	Z^R^Z^R^, Z^R^W	0	49.2 ± 25.1	85.8 ± 14.5	n.d.
CpRR1_F5	CpRR1_F5f × CpSm	8, 302	Z^R^Z^S^, Z^S^W	50	63.4 ± 15.9	87.2 ± 14.1	n.d.
	CpRR1_F5m × CpSf	11, 638	Z^R^Z^S^, Z^R^W	0	12.1 ± 8.9	57.4 ± 23.4	28.6 [2:5]
CpRR1_F7	CpRR1_F7f × CpSm	13, 518	Z^R^Z^S^, Z^S^W	50	16.0 ± 8.4	16.3 ± 13.1	41.2 [7:10]
	CpRR1_F7m × CpSf	8, 452	Z^R^Z^S^, Z^R^W	0	*3.8 ± 5.2	*4.1 ± 7.7	n.d.

^$^ based on the hypothesis of dominant Z-linked resistance inheritance in CpRR1, progeny genotypes and expected mortality (Exp. mort.) (%) at 7 dpi according to Asser-Kaiser et al [5]. * indicates that negative value of Abbott [20] corrected mortality in independent single-pair crossings (5 out of 8) were set as zero. “-” was not counted in the experiment. n.d., not determined because of fungal contamination at the pupal stage.
